# Case report of a transcatheter pulmonic valve-in-valve replacement through a bioprosthetic tricuspid valve in a patient with carcinoid heart disease

**DOI:** 10.1093/ehjcr/ytaf484

**Published:** 2025-10-06

**Authors:** Rachael Lyons, Frank Qian, Matthew H Kulke, Ashvin Pande, Omar S Siddiqi

**Affiliations:** Department of Medicine, Boston Medical Center, Boston University Chobanian & Avedisian School of Medicine, 715 Albany St. E-113, Boston, MA 02118, USA; Department of Medicine, Boston Medical Center, Boston University Chobanian & Avedisian School of Medicine, 715 Albany St. E-113, Boston, MA 02118, USA; Department of Medicine, Boston Medical Center, Boston University Chobanian & Avedisian School of Medicine, 715 Albany St. E-113, Boston, MA 02118, USA; Department of Medicine, Boston Medical Center, Boston University Chobanian & Avedisian School of Medicine, 715 Albany St. E-113, Boston, MA 02118, USA; Department of Medicine, Boston Medical Center, Boston University Chobanian & Avedisian School of Medicine, 715 Albany St. E-113, Boston, MA 02118, USA

**Keywords:** Case report, Carcinoid heart disease, Bioprosthetic valve replacement, Valve-in-valve replacement

## Abstract

**Background:**

Carcinoid heart disease (CHD) is a paraneoplastic syndrome that occurs in patients with metastatic neuroendocrine tumours (NETs), often resulting in right-sided valvular dysfunction and heart failure. Surgical valve replacement is the main treatment for CHD with valvular involvement, though many patients will eventually develop prosthetic valve degeneration.

**Case summary:**

We present a case of a 70-year-old female with metastatic NET, complicated by CHD status post-surgical tricuspid and pulmonary valve replacement who presented with dyspnoea. She was found to have worsening right-sided heart failure in the setting of severe bioprosthetic pulmonic valve regurgitation. Given the patient’s metastatic NET, prior sternotomy, and overall frailty, it was felt that surgical valve replacement would portend prohibitively high risks. In turn, the patient underwent transcatheter pulmonic valve-in-valve replacement via a bioprosthetic tricuspid valve. Post-deployment trans-oesophageal echocardiography showed good pulmonic valvular function with no paravalvular leak, reduction of PR to mild, and reduction in the mean gradient from 15 to 4 mmHg. There was no evidence of damage to the bioprosthetic tricuspid valve.

**Conclusion:**

This case demonstrates the feasibility, safety, and short-term efficacy of bioprosthetic valve-in-valve interventions in patients with CHD with bioprosthetic valve degeneration.

Learning points

- Surgical valve replacement is the main treatment for carcinoid heart disease with valvular involvement, though many patients will eventually develop prosthetic valve degeneration.
- Percutaneous valve replacement may be a safe and efficacious treatment for bioprosthetic valve degeneration in patients with carcinoid heart disease.

## Introduction

Carcinoid heart disease (CHD) is a paraneoplastic syndrome that occurs in patients with metastatic neuroendocrine tumours (NETs), often resulting in right-sided valvular dysfunction and heart failure. Surgical valve replacement is the mainstay treatment for CHD with valvular involvement, though many patients develop prosthetic valve degeneration. We discuss a case of successful transcatheter pulmonic valve-in-valve (ViV) replacement through a bioprosthetic tricuspid valve (TV) in a patient with metastatic NET and prior surgical tricuspid and pulmonic valve (PV) replacements, who presented with worsening right-sided heart failure in the setting of severe bioprosthetic PV regurgitation.

## Summary figure

**Figure:**
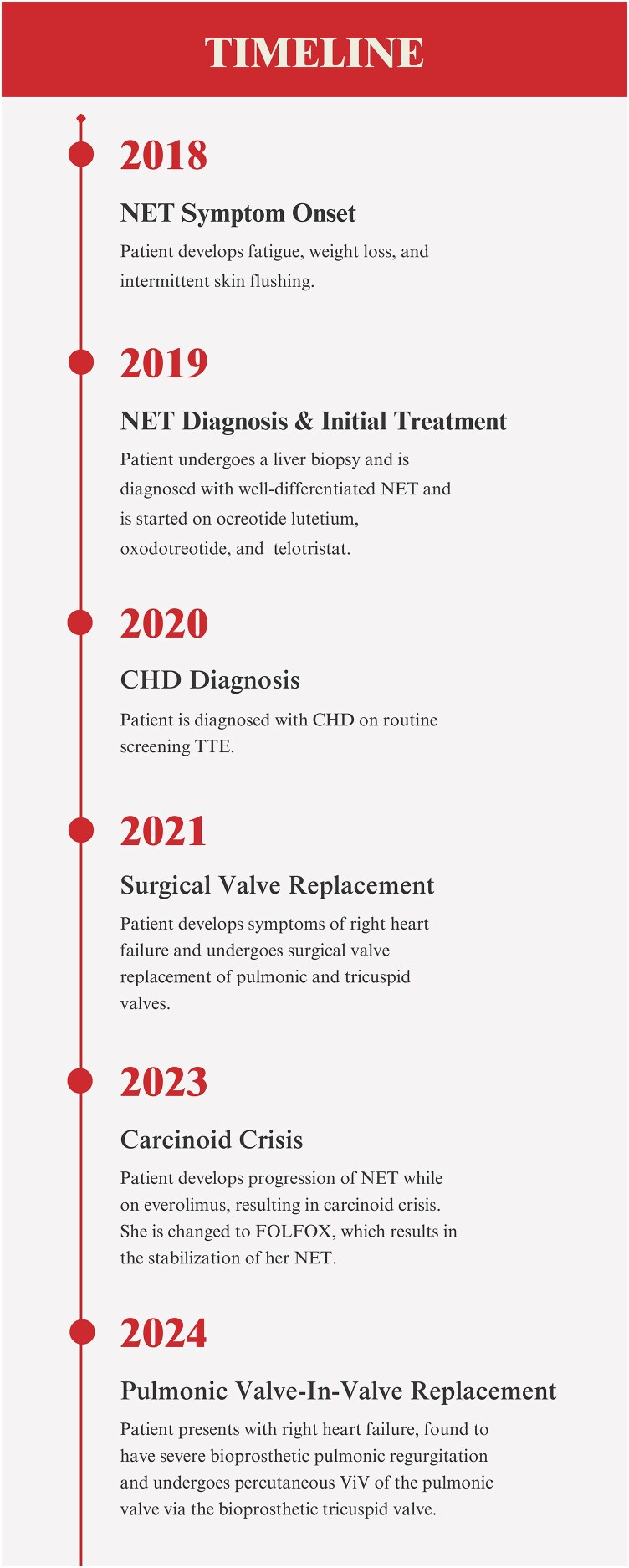


## Case presentation

A 70-year-old female with small bowel NET with metastases to the liver, complicated by CHD that was treated with surgical TV and PV replacements 3 years prior, presents with worsening dyspnoea on exertion. She was found to be in decompensated heart failure as evidenced by elevated jugular venous pressure, abdominal ascites, and pitting oedema in the bilateral lower extremities.

**Figure 1:**
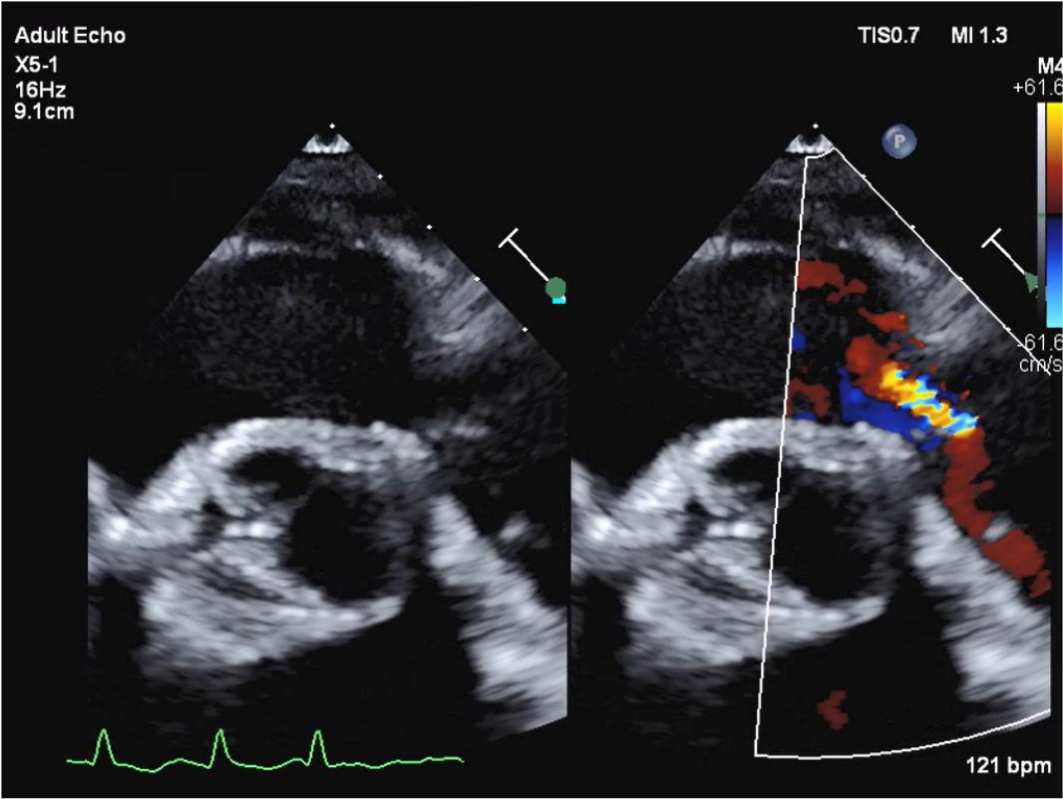
Transthoracic echocardiography, parasternal short-axis view, showing thickened and restricted prosthetic pulmonic valve leaflets resulting in severe pulmonary regurgitation.

**Figure 2:**
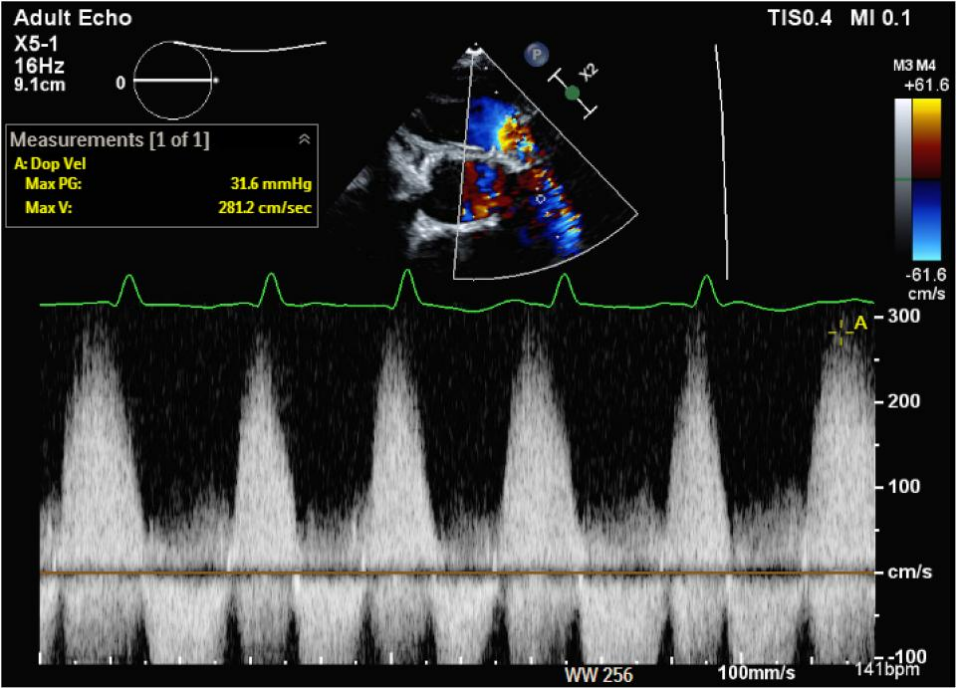
Continuous Doppler profile across the bioprosthetic pulmonic valve demonstrating a dense pulmonary regurgitant jet with a peak velocity of 2.8 m/s.

**Figure 3:**
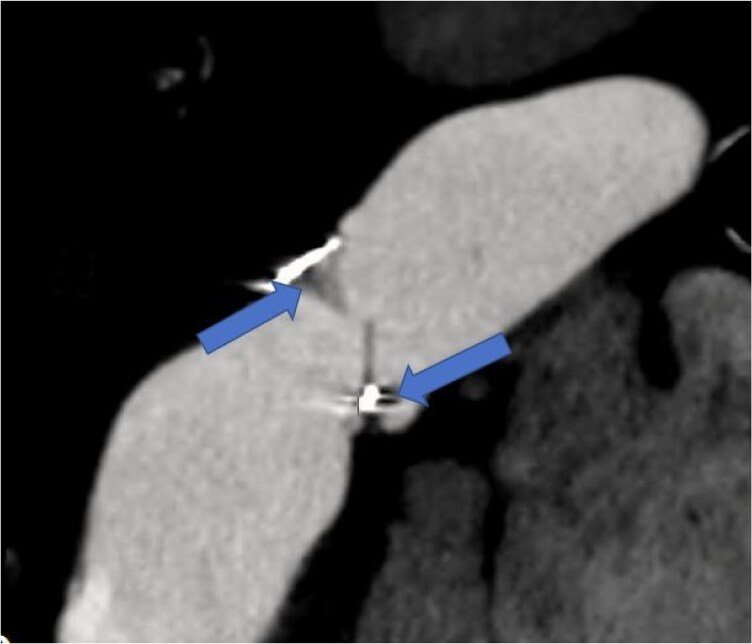
Cardiac computed tomography showing bioprosthetic pulmonic valve degeneration with pannus formation (arrows) on the leaflets resulting in significant mal-coaptation.

Her initial evaluation was notable for a B-type natriuretic peptide level of 701 pg/mL, elevated from her baseline of 180–200, and chromogranin A (CgA) of 16 292 ng/mL, elevated above the upper limit of normal (311 ng/mL) and her level following initial valve replacement (5605 ng/mL). Transthoracic echocardiography (TTE) showed normal left ventricular systolic function and a well-seated tricuspid prosthetic valve with moderate regurgitation. The right ventricle (RV) was significantly dilated with reduced systolic function with a TAPSE of 1.3 cm and a tricuspid S′ of 7.3 cm/s. The prosthetic PV was well seated with elevated antegrade gradients, restricted leaflets, and severe pulmonic regurgitation (PR). Severe PR was quantified based on a pressure half-time of the PR jet of 70 ms and a PR volume of 44 mL right ventricular outflow tract stroke volume of 76.9 mL, LVOT stroke volume of 32.9 mL), with a regurgitant fraction of 57% (*Figures 1* and *2*). A cardiac computed tomography showed thickened bioprosthetic PV leaflets with severe mal-coaptation due to reduced leaflet excursion during systole with evidence of mild pannus formation (*Figure 3*). A right heart catheterization demonstrated elevated right-sided pressures with right atrial pressure of 8 mmHg, pulmonary pressures of 34/10 mmHg with a mean of 17 mmHg, pulmonary capillary wedge pressure of 11 mmHg, and low-normal cardiac index of 2.1 L/min/m^2^ by the Fick equation. The peak gradient across the PV was 15 mmHg, consistent with mild stenosis. Ultimately, the patient’s RV failure was thought to be secondary to bioprosthetic PV degeneration.

A multi-disciplinary heart team comprising cardiothoracic surgery, interventional cardiology, cardiac anaesthesia, cardio-oncology, and medical oncology was convened. Given the patient’s metastatic NET, heart failure, prior sternotomy, malnutrition, and overall frailty (5 on the Rockwood Frailty Scale), surgical replacement of the bioprosthetic PV was of prohibitive risk. After shared decision-making discussion with the patient and her oncology team who felt that the NET was manageable, we elected to proceed with transcatheter ViV replacement.

**Figure 4:**
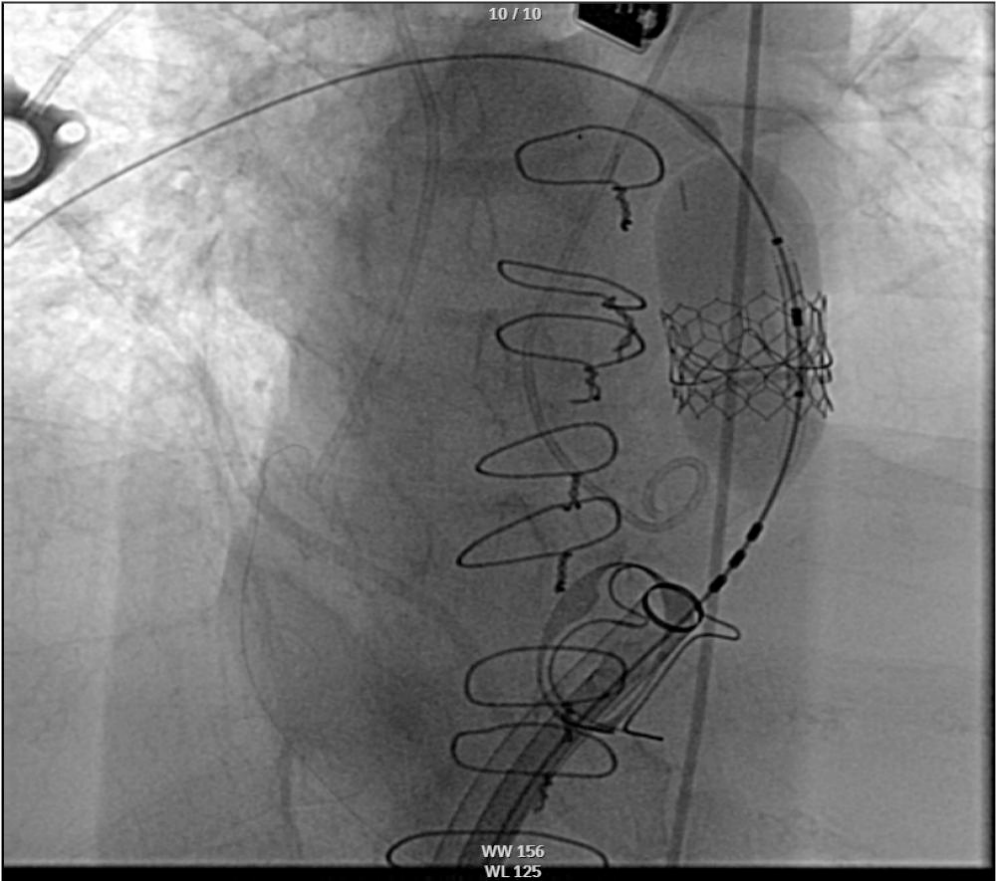
Fluoroscopy image showing deployment of the 23 mm Edwards SAPIEN transcatheter valve within the prior bioprosthetic pulmonic valve.

**Figure 5:**
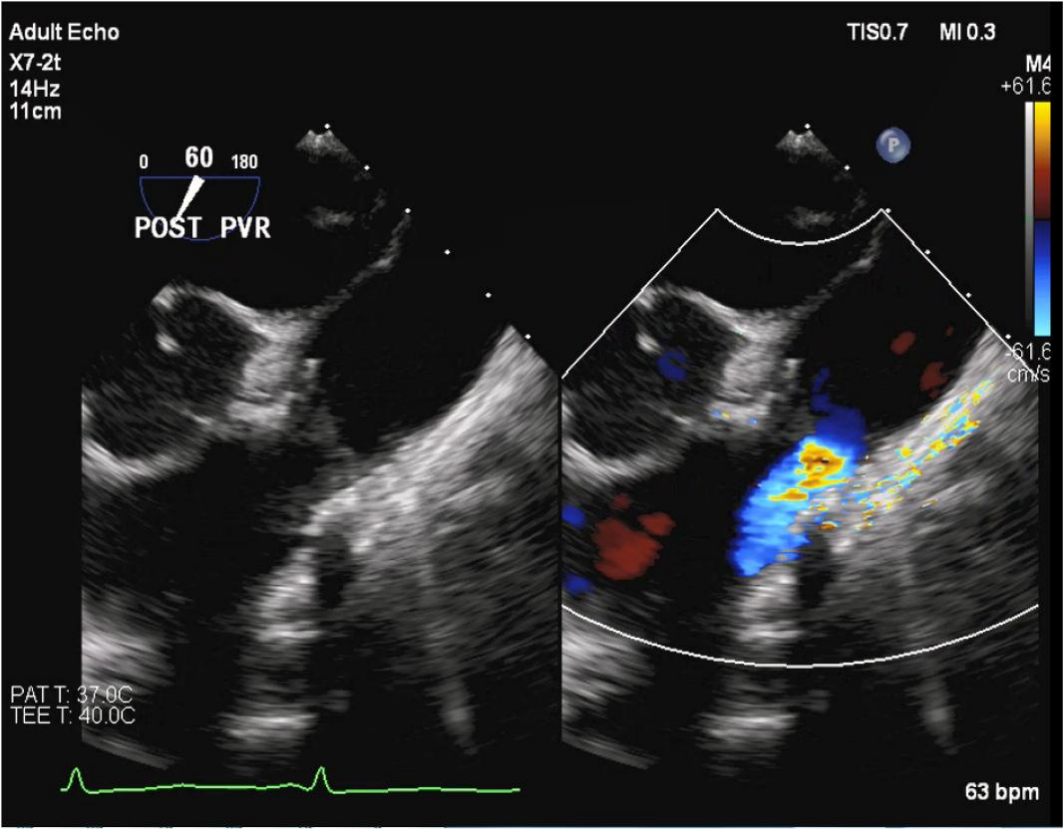
Intra-operative trans-oesophageal echocardiography, mid-oesophageal short-axis view at 60°, showing reduction in the pulmonic regurgitation to mild post-transcatheter valve-in-valve intervention.

The patient was admitted to the hospital 24 h prior to the procedure for volume optimization. Given the intra-operative risk of carcinoid crisis, she was prophylactically treated with a onetime dose of octreotide 500 mcg. The patient was placed under general anaesthesia, and bilateral femoral venous and left femoral arterial access was obtained. A 5-Fr pigtail catheter was placed in the aortic root for visualization of coronary course around the PV. The sheath was placed over a Lunderquist Double-Curved Extra-Stiff guidewire (Cook Medical) 24-Fr Gore DrySeal with the tip across the prosthetic PV. Subsequently, a 23 mm Edwards SAPIEN S3 valve was introduced and brought into position within the pulmonic prosthesis and deployed at nominal volume (*Figure 3*). Post-deployment trans-oesophageal echocardiography showed good valvular function with no paravalvular leak, reduction of PR to mild, and reduction in the mean gradient from 15 to 4 mmHg (*Figures 4* and *5*). The TV appeared unchanged with stable moderate TR.

The patient tolerated the procedure well, reported immediate improvement in exercise capacity, and was discharged from the hospital the next day. A TTE performed ∼2 months post-op showed a well-functioning prosthetic pulmonic ViV with a peak gradient across the ViV pulmonic prosthesis of 17 mmHg with a mean gradient of 10 mmHg. These values corresponded to a calculated effective valve orifice area of 1.6 cm^2^ (1.0 cm^2^/m^2^ when indexed to body surface area). There was also preserved function of the bioprosthetic TV with a trans-valvular velocity of 1.4 m/s with a mean gradient of 4 mmHg at a heart rate of 61 b.p.m. At the 2-month follow-up appointment, the patient reported marked improvements in her ability to perform daily activities and was able to take an international trip.

## Discussion

Carcinoid heart disease is a paraneoplastic syndrome resulting from 5-hydroxytryptamine (serotonin) and other bioactive hormones that are secreted by tumour cells and lead to plaque-like deposits on valvular cups. Serotonin is inactivated by enzymes found in the lung vasculature; thus, the right side of the heart is most often affected.^1^ Carcinoid heart disease portends a poor prognosis in patients with NET. Without valve replacement, patients with New York Heart Association class III or IV symptoms have a median survival of only 11 months. Those who undergo surgical valve replacement have a 35% survival rate at 5 years.^2^

Though surgical valve replacement is considered a definitive treatment for CHD, in rare cases, patients with CHD who are at high risk for sternotomy have undergone percutaneous valve replacement as an initial or a subsequent treatment.^3,4^ This is the only case, to our knowledge, of a CHD patient undergoing percutaneous pulmonic ViV replacement through a bioprosthetic TV, without simultaneous tricuspid ViV replacement. This presents several unique technical challenges. First, the presence of a prosthetic TV may hinder appropriate positioning and deployment of the PV. Compared to the prior cases of simultaneous tricuspid and pulmonic ViV replacements for severe TR and severe PR, in the case of our patient with moderate TR and severe PR, one notable challenge was to navigate the bioprosthetic TV with our sheath without compromising the existing bioprosthetic TV structure and in turn worsening TR. The presence of significant RV remodelling in our patient further exacerbated the difficulties of valve deployment. Furthermore, in general, plaques characteristic to CHD distort the anatomy of the bioprosthetic valve, making it difficult to position the valve and create an adequate seal during deployment. Finally, compared to native percutaneous valve replacements, ViV replacement presents added challenges in sizing, positioning, and implanting the valve, which increases the risk of complications such as coronary obstruction, valve migration, and high residual gradients. Our case demonstrates the feasibility of pulmonic ViV replacement via a prosthetic TV in CHD, despite the many technical challenges. There have been limited long-term outcomes data on individuals who undergo a transcatheter pulmonic ViV intervention. Extrapolation from data on individuals who undergo repeat transcatheter pulmonic interventions, primarily due to congenital heart disease, shows favourable outcomes with the cumulative incidence of surgical explant after intervention, being 13.4% at 5 years overall and 7.7% among patients whose reintervention was for an indication other than endocarditis.^5^

## Conclusion

We present a case of transcatheter PV in valve replacement through a bioprosthetic TV in a patient with stable metastatic NET and CHD who presented with worsening right-sided heart failure in the setting of severe bioprosthetic PR.

## Lead author biography


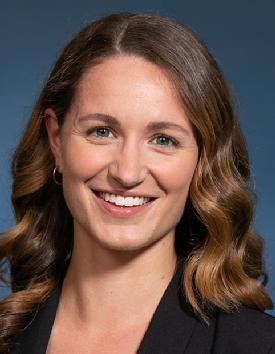


Rachael Lyons is a second year Internal Medicine Resident at Boston Medical Center/Boston University in Boston, Massachusetts. She plans to pursue a career in preventive cardiology. She enjoys long distance running, cycling, and cooking.

## Supplementary Material

ytaf484_Supplementary_Data

## Data Availability

The data underlying this article will be shared on reasonable request to the corresponding author.

## Notes

**Consent:** The authors confirm that a written consent for submission and publication of this case report including images and text was obtained from the patient in line with the COPE guidelines.

**Funding:** None declared.

## References

[1] Jin C, Sharma AN, Thevakumar B, Majid M, Al Chalaby S, Takahashi N, et al Carcinoid heart disease: pathophysiology, pathology, clinical manifestations, and management. Cardiology 2020;146:65–73. 33070143 10.1159/000507847

[2] Connolly HM, Schaff HV, Abel MD, Rubin J, Askew JW, Li Z, et al Early and late outcomes of surgical treatment in carcinoid heart disease. J Am Coll Cardiol 2015;66:2189–2196. 26564596 10.1016/j.jacc.2015.09.014

[3] Khan JN, Doshi SN, Rooney SJ, Bhabra MS, Steeds RP. Transcatheter pulmonary and tricuspid valve-in-valve replacement for bioprosthesis degeneration in carcinoid heart disease. Eur Heart J Cardiovasc Imaging 2016;17:114. 26497736 10.1093/ehjci/jev279

[4] Luthra S, Olevano C, Richens T, Tsang GM. Percutaneous transcatheter valve-in-valve pulmonary and tricuspid replacement in carcinoid heart disease. JACC Case Rep 2020;2:533–536. 34317287 10.1016/j.jaccas.2019.11.089 PMC8298683

[5] McElhinney DB, Zhang Y, Levi DS, Georgiev S, Biernacka EK, Goldstein BH, et al Reintervention and survival after transcatheter pulmonary valve replacement. J Am Coll Cardiol 2022;79:18–32. 34991785 10.1016/j.jacc.2021.10.031

